# The role of mobile health technologies in promoting COVID-19 prevention: A narrative review of intervention effectiveness and adoption

**DOI:** 10.1177/20552076221131146

**Published:** 2022-10-20

**Authors:** Jane Katusiime, Wilson Tumuhimbise, Godfrey Rwambuka Mugyenyi, Phionah Kobutungi, Aaron Mugaba, Raphael Zender, Niels Pinkwart, Angella Musiimenta

**Affiliations:** 1 Department of Computer Science, 9373 Humboldt Universität zu Berlin, Berlin, Germany; 2 Faculty of Computing and Informatics, 108123 Mbarara University of Science and Technology, Mbarara, Uganda; 3 Faculty of Medicine, 108123 Mbarara University of Science and Technology, Mbarara, Uganda

**Keywords:** coronavirus disease 2019 prevention, mHealth, technology adoption, intervention effectiveness

## Abstract

**Background:**

Researchers have found innovative ways of using mobile health (mHealth) technologies to prevent the spread of coronavirus disease 2019 (COVID-19). However, fewer studies have been done to determine their adoption and effectiveness.

**Objective:**

This review summarises the published evidence on the effect of mHealth technologies on the adoption of COVID-19 preventive measures, prevention knowledge acquisition and risk perception as well as technology adoption features for COVID-19 prevention.

**Methods:**

PubMed, IEEE and Google Scholar databases were searched for peer-reviewed literature from 1 January 2020 to 31 March 2022 for studies that evaluated the effect of mHealth technologies on COVID-19 preventive measures adoption, prevention knowledge acquisition and risk perception. Thirteen studies met the inclusion criteria and were included in this review. All the included studies were checked for quality using the mHealth evidence reporting and assessment (mERA) checklist.

**Results:**

The review found out that the utilisation of mHealth interventions such as alert text messages, tracing apps and social media platforms was associated with adherence behaviour such as wearing masks, washing hands and using sanitisers, maintaining social distance and avoiding crowded places. The use of contact tracing was linked to low-risk perception as users considered themselves well informed about their status and less likely to pose transmission risks compared to non-users. Privacy and security issues, message personalisation and frequency, technical issues and trust concerns were identified as technology adoption features that influence the use of mHealth technologies for promoting COVID-19 prevention.

**Conclusion:**

Utilisation of mHealth may be a feasible and effective way to prevent the spread of COVID-19. However, the small study samples and short study periods prevent generalisation of the findings and calls for larger, longitudinal studies that encompass diverse study settings.

## Introduction

Coronavirus disease 2019 (COVID-19) was first discovered in Wuhan, China, in December 2019 and quickly spread across the world. It was announced as a global pandemic by the director general of World Health Organisation on 11 March 2020.^1^ There has since been a mutation of the coronavirus and emerging of new variants such as Beta, Gamma but Delta and Omicron have been considered to spread quickly making them variants of concern.^2^ Globally, there were 452,201,564 confirmed cases of COVID-19 including 6,029,852 deaths as of 12 March 2022.^3^ The first case of COVID-19 in Africa was recorded on 14 February 2020 in Egypt.^4^ As of 14 March 2022, 47 African countries have been affected by the pandemic with 8,139,831 cumulative cases and 169,722 deaths.^5^

As a preventative measure, many countries imposed lockdowns characterised by the closure of schools, businesses and organisations, ban of private and public transport and curfews. Much as these lockdowns may have reduced the spread of the virus to some extent, they have created social economic and public health challenges.^6,7^ To begin with, health facilities especially in low-resource settings (LRS) have limited intensive care units and the associated resources to deal with critical cases. The presence of COVID-19 critical cases has resulted in the worsening of these shortages and high treatment costs which have negatively affected non-COVID-19 patients in need of intensive care.^8^ Similarly, focus has been put on how to treat and control COVID-19 cases leading to less emphasis on other public health programmes such as routine vaccinations for other diseases and treatment of other health conditions such as non-communicable diseases.^9^ This may lead to outbreaks of other diseases and worsen the already worrying public health issues.

The pandemic has negatively affected maternal and child health due to travel limitations that limit access to health facilities and social support.^10,11^ Studies carried out on pregnant women in Nepal, Italy, China and India reported mental health issues such as feelings of anxiety and depression resulting from fear of mother-to-child COVID-19 transmission, limited access to antenatal care and limited social support.^11–14^ Health practitioners in Uganda, Kenya and Tanzania reported low numbers of women accessing antenatal care due to fear of testing positive for COVID-19 and being isolated from families, lack of financial resources and limited transport options due to lockdowns.^15^ This has led to women giving birth without skilled assistance or going to hospitals when it is too late, which has resulted in undesired outcomes such as maternal deaths and stillbirths.^15^

Despite the discovery of the vaccine, people living in LRS have limited access to the vaccines. For instance, as of February 2022, Uganda had administered 16.7 million vaccine doses out of the required 66 million doses with only 7.7 million people fully vaccinated out of the 33 million people to be vaccinated.^16,17^ Consequently, LRS mainly rely on public health prevention measures such as lockdowns, social distancing, wearing masks and washing hands.^2^ COVID-19 prevention information is being disseminated using various channels such as mass media (radios, television and newspapers), dedicated social network sites and websites.^18^ Frequent advertisements on televisions and prominent social media platforms such as Facebook and Instagram encouraging people to social distance, stay home among other prevention measures provided a platform for reaching out to and delivering prevention information to a wide audience.^19^ The repetitive display of print media on the prevention of COVID-19 in newspapers and public spaces such as supermarkets, public transport may have played an essential role in reducing the spread of the virus.^19^ Trusted public health organisation websites such as World Health Organisation^20^ and Centre for Disease Control^21^ provided trusted information on the pandemic.^22^

Given the widespread telephone ownership and mobile network coverage especially in LRS, mobile phone–based technologies can potentially be used as alternative or supplementary channels to disseminate COVID-19 prevention information and promote the prevention of the pandemic. For instance, penetration of mobile phones in Uganda stood at 68%^23^ and Nigeria at 85% as of 2019.^24^ This technology boom has created opportunities in health care leading to emerging of mobile health (mHealth) innovations in various health fields such as maternal healthcare,^25–27^ tuberculosis (TB) care^28^ and management of pandemics such as COVID-19.^29^ mHealth technologies may offer low-cost approaches for accessing healthcare services in rural settings like Uganda^30^; however, literature documenting their utilisation for COVID-19 prevention remains limited. The use of low-cost mHealth technologies (such as emergency text messages, trackers and chatbots) to disseminate COVID-19 information, track and notify contacts of COVID-19 patients have resulted in acquisition of COVID-19 prevention knowledge and adoption of preventive measures.^3,29^ The WHO Health alert implemented via WhatsApp and Facebook disseminated COVID-19-related facts/information in various languages to a wide audience which can potentially facilitate knowledge acquisition and reduction of misinformation.^31^ The use of SwissCovid app to track and notify contacts of COVID-19 patients enabled the notified contacts to follow up with calls to designated call centres and seek guidance and access primary care when necessary.^32^ However, knowledge about the effectiveness of mHealth technologies for preventing COVID-19 is limited as many of the existing studies remain unevaluated. Also, little is known about the mHealth features that influence their usage. This narrative review evaluates the effectiveness of mHealth interventions in promoting COVID-19 prevention (i.e. effect on the adoption of preventive measures, prevention knowledge acquisition and risk perception). The narrative also highlights mHealth features that influence the use of these technologies for promoting COVID-19 prevention.

## Methods

### Search strategy

This review focuses on the literature that covers the effects of mHealth interventions on COVID-19 prevention. The searches were conducted in MEDLINE (PubMed), IEEE and Google Scholar using search strings as follows:

Google scholar: “Mobile Health” OR mHealth Technologies OR Applications AND “COVID-19” Prevention

Pubmed: (((((“Mobile Health”[All Fields] AND (“technology”[MeSH Terms] OR “technology”[All Fields] OR “technologies”[All Fields] OR “technology s”[All Fields])) OR (“phone s”[All Fields] OR “phoned”[All Fields] OR “phones”[All Fields] OR “phoning”[All Fields] OR “telephone”[MeSH Terms] OR “telephone”[All Fields] OR “phone”[All Fields]) OR (“smart mater struct”[Journal] OR “sms”[All Fields])) AND “COVID-19”[All Fields]) OR (“corona”"[All Fields] OR “coronae”[All Fields] OR “coronas”[All Fields])) AND “Prevention”[All Fields]) AND (2020:2021[pdat])

IEEE Xplore: (“All Metadata”:Mobile Health) AND (“All Metadata”:COVID19) OR (“All Metadata”:Corona) AND (“All Metadata”:prevention). Keywords were determined with guidance from various search strategies of related mHealth studies on COVID-19.

### Study selection

**Figure 1.**
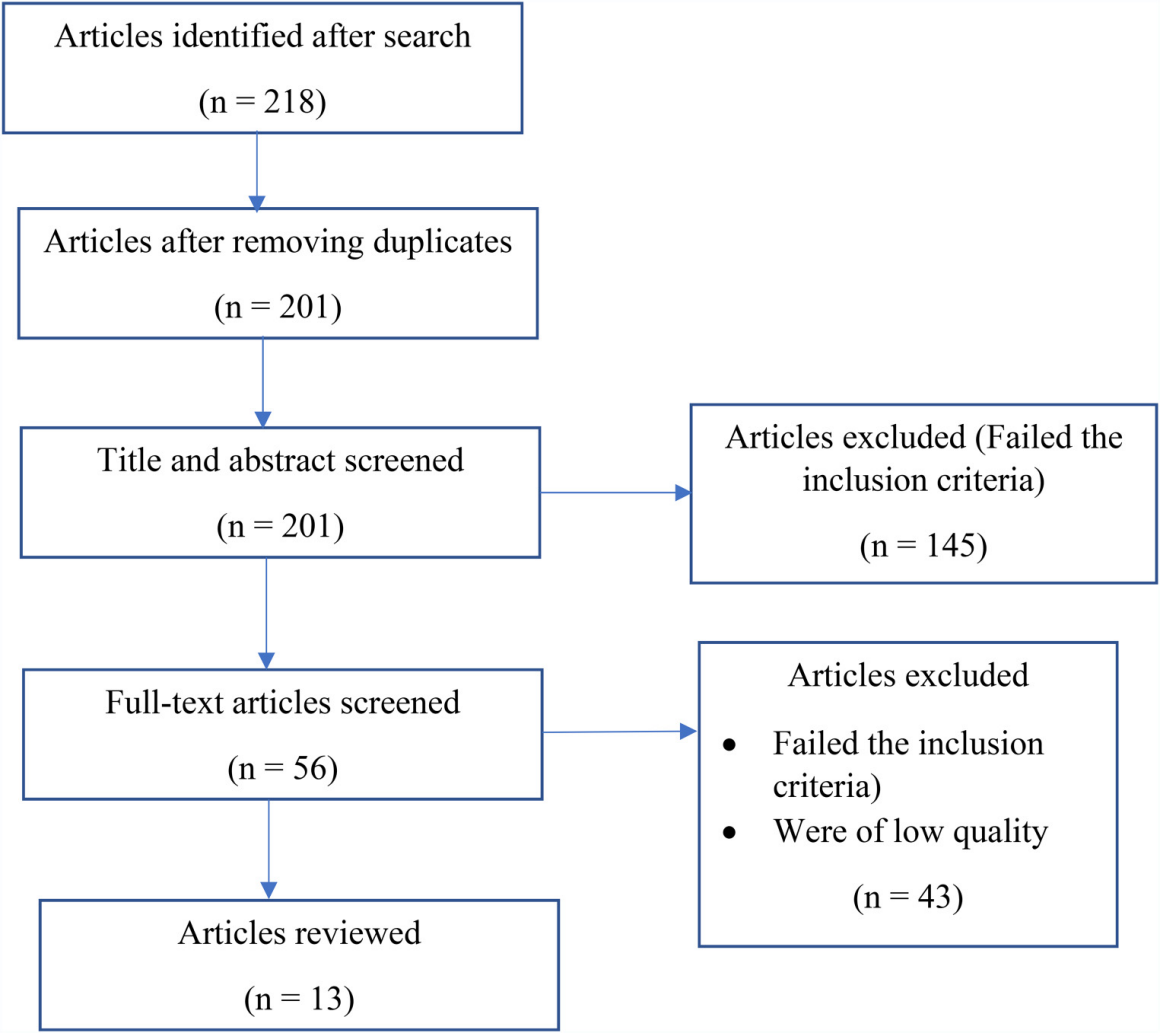
Overview of the selection process.

In total, we identified 218 articles from the searches which were reduced to 201 after eliminating the duplicates. The articles were then screened in two phases based on the inclusion and exclusion criteria, as shown in Table 1. In the first phase, the title and abstract were evaluated and 56 articles that were relevant were chosen. This first phase eliminated 145 articles. Reasons for this elimination include reporting on digital technologies other than mHealth (47%, 68/145), reporting on mHealth apps not related to COVID-19(36%, 52/145), reporting on mHealth technologies used in clinical settings only (11%, 16/145) and commentaries/opinions (6%, 9/145). In the second phase, the full texts of the chosen articles were screened and their content that included author and year, mHealth intervention, study design, general findings, effectiveness and adoption were extracted and tabulated. The contents were then evaluated against the eligibility criteria leading to the elimination of 43 articles (77%, 43/56). After this process, 13 articles were considered relevant and included in the review as shown in Figure 1.

**Table 1.** Inclusion and exclusion criteria.

Inclusion criteria
Studies that evaluated the effect of mHealth technologies on: the adoption of COVID-19 preventive measures, or/and acquisition of COVID-19 prevention knowledge or/and COVID-19 risk perception. Studies that reported on technology adoption features that influence use of mHealth technology for COVID-19 prevention. Studies that focused on patient-centred technologies. Studies written in English. Studies published between 2020 and 2021.
Exclusion criteria
Studies that reported on digital technologies other than mHealth. Studies on mHealth apps not related to COVID-19. Studies that reported mHealth technologies used in hospital/clinic settings only. Commentaries and opinions.

COVID-19: coronavirus 2019; mHealth: mobile health.

### Inclusion and exclusion criteria

Based on the objectives of the review, we derived the inclusion and exclusion criteria as shown in Table 1.

Quality assurance

All the articles included in the review were assessing the effectiveness of mHealth interventions and it was, therefore, important to assess the quality of the mHealth interventions reported in the studies. The quality of these interventions was assessed based on the mHealth evidence reporting and assessment (mERA) checklist,^33^ which follows 16 guidelines that include intervention content, adoption inputs, intervention delivery and technology platform. The guidelines were used because they have been tested and found to be reliable in guiding the development of reports on studies that evaluate the effectiveness of mHealth interventions.

All the included studies were assessed against the mERA checklist and met between 50% and 90% of the guidelines. All the included papers reported on the technology content, content delivery, technology design, technology platform and user feedback as captured under the description, delivery mode and effectiveness sections as shown in Table 2. All the studies that scored below 50% were considered to be of low quality and excluded.

**Table 2.** Key components of the reviewed articles.

No.	Author and country	Description of the Technology	Study design	Theory/Model	Delivery mode	Effectiveness/findings	mHealth features that influence adoption
1	You and Lee^34^ South Korea	Korean citizen participants receive persuasive and risk information text messages on COVID-19 in a two-month study	Cross-sectional web-based survey with 990 participants utilising anonymous online questionnaires	Social cognitive theory used to determine individual behaviours between personal, behavioural and environmental factors	Text messages	Improved adherence to wearing masks Avoidance of crowded places and social gatherings	Frequency of sending/receiving text messages (higher frequency led to lack of interest)
2	Boruchowicz et al.^35^ Brazil	Brazilian citizen participants receive informational, motivational and instructional SMS text messages in a three-week study	Randomised controlled trial with 75,351 participants that utilised telephone-based surveys to determine the impact of the intervention in promoting behavioural change during the pandemic	COM-B model to assess behavioural change	Text messages	Improved adherence to wearing masks Maintenance of the correct social distance Staying at home (avoidance of public places)	
3	Bahety et al.^36^ India	Respondents from Bihar, India received informative, fearful and pro-social motivational SMS text messages in Hindi in a two-month evaluation study	Adaptive randomised control trial with 3964 participants who utilised phone surveys to evaluate the impact of the intervention on the adoption of social distancing and hand washing		Text messages	No impact on adoption of social distancing and hand washing	SMS literacy (high literacy led to higher access to messages)
4	Sun et al.^37^ Italy, Spain, Denmark, the United Kingdom and the Netherlands	Social media: Italy, Spain, Denmark, the United Kingdom and the Netherland participants's use of social, dating and communication apps was monitored via their mobile phones and Fitbit devices in a two-week study	The quantitative study with 1062 participants used RADAR surveys to automatically capture data remotely		Social media apps	Adoption of physical distancing as a result of participants’ keeping active on the mobile devices	
5	Li and Liu^38^ China	Used social media apps such as WeChat and Ding Xiang Doctor to learn about news of COVID-19 in a nine-day study with Chinese internet users	Web-based cross-sectional study with 802 participants using structured questionnaires	Social cognitive theory was used to explain how people learn other people's behaviour through observing	Social media apps	Improved covering mouth and nose when sneezing or coughing Improved hand washing	
6	Almagor and Picascia^39^ Scotland	Contact tracing: CTA keeps IDs of agents and notifies their contacts of possible exposure if an agent tests positive within 10 days in a study with urban population of Glasgow, Scotland	Simulation-based study with 103,000 participants	An agent-based model was used to simulate the spread of COVID-19 within the population of an urban area	Contact tracing app	Self-isolation among alerted participants exposed to the virus	
7	Garrett et al.^40^ Australia	Contact tracing: CTA, telecommunication tracking and Bluetooth notification system used to track users in an 18-day study with 18 and above year olds from Australia	An online survey with over 4000 respondents		Smartphone tracing systems	Adherence to quarantine orders	Technical issues such as draining the battery, and using up mobile phone storage constrained intervention utilisation
8	Almalki^41^ Saudi Arabia	Health chatbot: used to disseminate health information about COVID-19 in a four-week study with 18 and above year olds from Saudi Arabia	Cross-sectional quantitative descriptive study with 173 participants, utilising online surveys		Health chatbot	Knowledge on how to wash hands and wear masks Knowledge of self-tracking COVID-19 symptoms	Privacy concerns such as collection of personal data (names, telephone contacts and location)
9	Suppan et al.^42^ Switzerland	Game: EscapeCOVID-19 game quizzes the user about COVID-19, a wards right answers with tokens and wrong answers with a virus. Its game over when the user accumulates 5 viruses. A one-month study was carried out with long-term care facility employees from Geneva, Switzerland	Randomised controlled trial using a web-based triple blind (investigator, participants, data analyst) survey with 652 participants	Nicholson's RECIPE (Reflection, Engagement, Choice, Information, Play, and Exposition) mnemonic	Game	Willingness to change prevention behaviour such as frequent change of gloves and practicing hand hygiene	
10	Tomczyk et al.^43^ Germany	Contact tracing: Corona Warn-App was used to track and alert contacts of COVID-19 patients using Bluetooth technology in a three-month study with participants from German population	Cross-sectional online survey with 349 respondents	Used UTAUT to assess the perceived usefulness and ease of use of a contact tracing app	Contact tracing app	Positive attitudes and fewer concerns about COVID-19 Lower frequency of COVID-19 experiences	Privacy and security features such as destroying data after 14 days Personalisation features of contact tracing apps such as provision of information tailored to individual needs
11	Altmann et al.^44^ France, Germany, Italy, the United Kingdom, and the United States	Contact tracing: CTAs were used to record user interactions and alert those at risk of contracting COVID-19 in a three-week study with 18 and above year olds from France, Germany, Italy, the United Kingdom, and the United States	Online survey with 5995 participants		Contact tracing app	An increase in feelings of anxiety and dislike for feedback about the possibility of infection	Trust of users in their governments
12	Camacho-Rivera et al.^45^ The United States	Contact tracing: mHealth tools were used to track potential COVID-19 exposure and symptoms, and provide recommendations in a three-month study of adults from the United States	Secondary data analysis of the COVID-19 impact survey among adults with health conditions		Contact tracing app	Enabled monitoring potential COVID-19 exposure, symptoms and recommendations	
13	Rodríguez et al.^46^ Spain	Contact tracing: RADAR COVID19 app was used to trace and alert close contacts of an infected individual in a one-month study with participants from San Sebastián de la Gomera- Canary Islands, Spain	Longitudinal population–based controlled experiment, and survey with 735 participants to assess the technical viability and the epidemiological impact of the app	Key performance indicator used to assess the potential usefulness of the app	Contact tracing app	Minimal follow-up by alerted contacts of COVID-19 patients (e.g. not calling designated point of care after being alerted)	Privacy and security features such as anonymity

COM-B: capability, opportunity, motivation, behaviour; COVID-19: coronavirus disease 2019; CTA: call-to-action; mHealth: mobile health; RADAR: remote assessment of disease and relapse; SMS: short message service; UTAUT: Unified Theory of Acceptance and Use of Technology.

## Results

### Effect of mHealth technologies on adoption of COVID-19 preventive measures

Seven (54%, 7/13) studies reported on the effect of mHealth technologies on the adoption of COVID-19 preventive measures. Of these, 3 (43%, 3/7) utilised text messages, 2 (29%, 2/7) utilised contact tracing technologies, while 2 (29%, 2/7) utilised social media platforms. You and Lee^34^ carried out a cross-sectional online evaluation of a short message service (SMS) text intervention implemented by the Korean government that included sending persuasive text messages to encourage citizens to adopt COVID-19 preventive measures, as well as texts containing risk information messages such as number of confirmed cases. The results of the study indicate that receiving COVID-19 emergency alert text messages was associated with adherence to wearing masks (76.8%, 760/990), washing hands and using sanitisers (72.1%, 714/990) and postponing or cancelling social events (63.4%, 628/990) in Korea.^34^ However, the effect of this two-month SMS text intervention on avoiding crowded places was less significant (57.7%, 571/990).

Boruchowicz et al.^35^ also conducted a randomised control trial of a text message–based intervention that involved sending a series of four text messages that informed, motivated or instructed participants to take up COVID-19 preventive measures. The three-week follow-up telephone survey–based study with 75,351 Brazilian citizens reported adherence to wearing masks when in public (77%) and maintaining the correct social distance when with other people (12.75% more likely by participants that received messages).^35^

On the other hand, Bahety et al.^36^ carried out an adaptive randomised control trial with 3964 respondents from rural Bihar, India, that involved sending neutral/informative, fearful and pro-social motivational text messages in Hindi to respondents. The results of this two-month SMS-based intervention showed no statistically significant results on hand washing (effect of 3.4 percentage points off of a control mean of 32% for the hand washing arm) and social distancing (0.3 percentage points off of 36% for the social distancing arm).^36^

A quantitative study with 1062 participants from five European countries (Italy, Spain, Denmark, the United Kingdom and the Netherlands) who were actively using social apps (social, dating and communication apps) such as Facebook, WhatsApp and Instagram was carried out.^37^ The study utilised remote assessment of disease and relapse (RADAR) surveys to automatically collect participants’ data (such as time spent at home, maximum number of Bluetooth-enabled devices nearby, phone unlock duration and time spent using the social apps) throughout the day via their mobile phones and Fitbit devices. The results of this two-week intervention show a positive effect on physical distancing/sociality denoted by (*p* < 0.001) between before lockdown and during lockdown in the five countries.

A web-based cross-sectional study with 952 Chinese internet users who were using social media apps (such as WeChat, People's Daily, Tencent News and Ding Xiang Doctor) was conducted in China. The participants were sent questionnaires to evaluate their usage of social media and its impact on their COVID-19 preventive behaviours, disease knowledge and health literacy. This nine-day (13–21 February 2020) study reported the adoption of public preventive behaviours (mean score 4.30/5, standard deviation (SD) 0.44), such as covering mouth and nose with tissue or sleeve when sneezing or coughing, and washing hands after going home during the COVID-19 pandemic.^38^

On the other hand, Almagor and Picascia^39^ conducted a simulation-based study with 103,000 over 14 years old, urban population participants (agents) from Glasgow, Scotland that involved the simulation of 140 scenarios with each repeated 20 times. The simulation utilises a contact tracing app that enables infected participants that have tested COVID-19 positive to notify their contacts of possible exposure. Results indicate that the use of the contact tracing app by 80% of the population reduced the infection rate from 45% to 15% and cases at the peak of the pandemic reduce by 89%. In addition, the use of the contact tracing app was associated with self-isolation among alerted contacts with a percentage mean of self-isolation of 70%.^39^

Furthermore, online surveys with over 4000 participants aged 18 years or older were conducted in Australia by Garrett et al.^40^ In this study, the Australian government among other technologies utilised telecommunication networks to do mandatory tracking of users, issue and enforce quarantine orders with fines and arrests when necessary. It further utilised a contact tracing app and a Bluetooth notification system. The results of this 18-day (6–23 June 2020) study show a reduction in contraction and spread of the coronavirus with posterior means of approximately 1.2 and 1.5, respectively.^40^

### Effect of mHealth technologies on COVID-19 prevention knowledge

Three (23%, 3/13) studies reported the effect of mHealth technologies on COVID-19 prevention knowledge. Of these, 1 (33%, 1/3) utilised health chatbots, 1 (33%, 1/3) utilised a game, while 1 (33%, 1/3) utilised text messages.

A cross-sectional, quantitative descriptive study with 173, over 18-year-old participants from Saudi Arabia that involved the use of health chatbots to disseminate health information about COVID-19 and to promptly answer users’ inquiries based on information sources was carried out.^41^ The results of this four-week online survey study show that 82.5% of the participants use chatbots to learn how to prevent COVID-19 (e.g. how to wear masks and how to wash hands), 82% use them to seek general information about COVID-19, 81% to learn about COVID-19 symptoms, 79% to look for nearby health services such as testing centres, 76% to self-assess COVID-19 symptoms and 72% to learn about COVID-19 medical treatments.

Suppan et al.^42^ conducted a randomised controlled trial with 652 participants from long-term care facility employees from Geneva, Switzerland, that involved evaluation of a web-based game (EscapeCOVID-19 game) that was used to test and educate the participants about COVID-19 through a question and answer strategy. Whenever a player chose an answer that can lead to infection, a red virus image appears but when the player selects an answer that fits a desirable behaviour, a positive token (thumbs-up image) is awarded. The game has a virus counter that incrementally increases and if the player gets a total of five viruses in the counter, a ‘game over’ screen appears. Results of this one-month study that utilised questionnaires indicate that 62% (74/119) of the participants acquired knowledge about face mask handling, protection of one's self from asymptomatic people and workplace disinfection from the information provided in the game and were willing to change their prevention behaviour as a result.^42^

In the same study by Boruchowicz et al.^35^ discussed in the previous section, receiving motivational, informational or instructional messages was associated with knowledge acquisition. Participants who received the civic duty messages were 13% more likely to report the right social distance to maintain from others and 3% more likely to wear a mask correctly compared to those who did not receive text messages.

### Effect of mHealth technologies on COVID-19 risk perception

Four (31%, 4/13) studies reported on the effect of mHealth technologies on COVID-19 risk perception. All of the four studies utilised contact tracing technologies. Tomczyk et al.^43^ conducted a cross-sectional online survey with 349 participants from the German population to assess the adoption and effect of contact tracing apps on COVID-19. This three-month study indicated that the use of apps such as Corona Warn-App to track and alert contacts of COVID-19 patients using Bluetooth resulted in fewer concerns about COVID-19 (anticipatory anxiety, *p* < 0.001with a mean (SD) of 3.41 (0.94)), and lower frequency of COVID-19 experiences (exposure- *p* < 0.001 with a mean (SD) of 54 (80.6)) among its users as they considered themselves well informed about their status and less likely to pose transmission risks compared to non-users.^43^

Furthermore, Altmann et al.^44^ conducted a cross-country study with 5995 over 18-year-old participants from France, Germany, Italy, the United Kingdom and the United States. This study utilised online surveys and involved use of contact tracing apps to record user interactions and alert those at risk of contracting COVID-19. This three-week (20 March–10 April 2020) study reported that use of the apps was linked to an increase in feelings of anxiety and dislike for feedback about the possibility of infection (26%, 1559/5995), concerns about government surveillance after the pandemic (42%, 2518/5995) and cybersecurity issues (35%, 2098/5995).^44^

A secondary data analysis study of the COVID-19 household impact survey was done by the National Opinion Research Centre at the University of Chicago, United States.^45^ The analysis considered three datasets collected from 10,760 adult (aged ≥ 18 years) participants between April and June 2020 but focused on mHealth tools that track potential COVID-19 exposure and symptoms and provide recommendations. Results of this three-month study indicate that the use of COVID-19 apps resulted in monitoring potential COVID-19 exposure, symptoms and recommendations among adults with mental health conditions, obesity and chronic health conditions (denoted by *p* ≤ 0.005) because the participants perceived themselves to be at a higher risk of contracting COVID-19.

Furthermore, Rodríguez et al.^46^ conducted a longitudinal population-based controlled experiment with 735 participants from San Sebastián de la Gomera, Canary Islands, Spain, that involved the evaluation of a Bluetooth-based digital contact tracing app (RADAR COVID19) that alerts close contacts of an infected individual. This one-month experiment was linked to minimal follow-up (10%) (e.g. call to designated point of care) by the notified close contacts of COVID-19 patients because the participants did not consider themselves to be at risk.^46^

### Technology adoption features that influence the use of mHealth technologies for promoting COVID-19 prevention

There are a number of technology features that influence users’ adoption of mHealth technologies for prevention of COVID-19 and we have categorised them as follows:

*Privacy and security*. Adoption of contact tracing apps was influenced by (1) privacy and security features such as anonymity and destroying data after 14 days in a cross-sectional online survey with German participants,^43^ in a controlled experiment with participants from Canary Islands, Spain^46^ and in an online survey with respondents from Australia.^40^ For instance, the RADAR COVID-19 app^46^ was developed following the privacy-by-design principles, which emphasise user anonymity and minimisation as stipulated by European legal standards. These strict privacy-preserving measures made an accurate estimation of the key performance indicators (KPIs) difficult and made gauging their uncertainty sometimes impossible.

On the other hand, the adoption of health chatbots was influenced by privacy concerns such as collection of personal data (names, telephone contacts and location) which hindered the effective deployment of health chatbots in a quantitative descriptive study with individuals from Saudi Arabia.^41^ Anonymity of users and destruction of collected data after sometime may be a solution to such hindrances, especially in situations where sensitive personal information is required.

Some studies emphasise the need for privacy/security in mHealth applications that capture sensitive health information.^47–49^ Much as privacy/security is crucial in mHealth applications, it is important to note that there is usually a trade-off between privacy/security and usability that has to be addressed. Applications that are very secure are less usable and vice versa.

*Message personalisation and frequency.* Frequent sending/receiving of text messages resulted in users not reading the messages in a cross-sectional study with Korean-speaking adults resident in Korea.^34^ Over 70% of the participants reported not reading messages because they were sent very often. On the other hand, personalisation features of contact tracing apps such as the provision of information tailored to individual needs were linked to the adoption intentions of the users in a cross-sectional online survey with German participants, especially among younger participants.^43^ The comfort of the users in terms of when and how often information is provided, and how it is packaged plays an important role in the usability and acceptability of mHealth apps. This is in line with studies in the literature that confirm the impact of personalisation such as feelings of empowerment, and keeping the patients engaged, which influence the adoption of mHealth apps.^50,51^

*Technical issues.* Technical issues regarding the use of contact tracing app such as draining the battery, and using up mobile phone storage were associated with refusal to download and use the apps, or uninstalling the apps in surveys with over 4000 adults in Australia.^40^ Participants who refused to download the smartphone tracking app reported battery usage as one of the hindrances. Users are dependent on mobile devices to maintain contact with social networks, monitor/manage health, carry out transactions and entertainment among other things. Any applications that threaten this through heavy utilisation of mobile device resources (storage, battery and internet) may face resistance from the possible user. This is also echoed by two studies that report the association of low-resource consumption of mHealth apps to their usability and acceptability.^49,52^

*Trust concerns.* Adoption of contact tracing apps was influenced by the trust of users in their governments in an online survey with 5995 participants from France, Germany, Italy, the United Kingdom and the United States.^44^ People who completely trusted the government were 25.9% more likely to download and install the app than those who had no trust in the government. Similarly, the official launch of the app with government support was associated with frequent use and app acceptance in a cross-sectional online survey with German participants.^43^ Studies confirm that trust in the source of the apps and government involvement plays an important role in the adoption and acceptance of the apps.^53,54^

## Limitations of the reviewed studies

Studies were limited in their study designs, for instance, 9 studies (69%, 9/13) reviewed relied on self-reporting which might have affected the findings due to the possibility of social desirability bias. For instance, a study by Camacho-Rivera et al.^45^ used self-reported data for the history of chronic health conditions instead of using medical records. Another study by Tomczyk et al.^43^ utilised a self-administered survey to report adoption intentions and the use of a contact tracing app.

The majority of the studies (77%, 10/13) were carried out in high-resource settings. For instance, the studies by Altmann et al.,^44^ Camacho-Rivera et al.,^45^ Li and Liu^38^ and Tomczyk et al.^43^ chose participants from western industrialised countries; participants aged ≥18 years; educated participants with a high income and aged 20–60 years; and participants from a German sample, respectively. These samples may not have been representative of the whole population thus making it unrealistic to generalise the findings.

Other studies had small sample sizes making it inappropriate to generalise the findings and difficult to determine the effect of larger samples on the results. For instance, the study by Garrett et al.^40^ had a sample size of a maximum of 1777 participants compared to total population of 25.7 m and a study by Boruchowicz *et al*.^35^ with a sample size of 75,351 participants out of a population of 212.6 m that both reported the need for scaling up the intervention to determine the real impact.

The majority of the studies (85%, 11/13) were cross-sectional making it difficult to longitudinally link attributes such as attitudes, intentions and behavioural changes. The average follow-up period of the studies was short (one month) making it hard to determine whether longer study periods might have an impact on the findings. Furthermore, a good number of studies (46%, 6/13) did not use a technology adoption and acceptance model/theory, which makes their findings unreliable.

## Conclusion

This review shows that the use of mHealth technologies such as contact tracing apps, health chatbots and text messages may be a feasible and effective way to prevent the spread of COVID-19. Our findings are in tandem with several reviews^55,56^ that have been carried out to assess the role of mHealth technologies in the prevention of infectious diseases like TB. Technology adoption features, such as personalisation of messages, privacy and security of information and users also influence adoption of the mHealth interventions. Despite these positive findings, the small study samples and short study periods prevent generalisation of the findings and calls for larger, longitudinal studies that encompass diverse study settings.
